# Extended susceptibility testing for refractory *Helicobacter pylori* infection: regional testing should guide antimicrobial decision making

**DOI:** 10.1186/s12879-025-12236-z

**Published:** 2025-12-16

**Authors:** Maria Paroikaki, Harin Navalan, Scott J. C. Pallett, Nabeela Mughal, Frances Davies, Saleh A. Alqahtani, Luke S. P. Moore

**Affiliations:** 1 Imperial College School of Medicine, Imperial College London South Kensington Campus, London, SW7 2AZ UK; 2 Clinical Infection Department, Chelsea and Westminster Hospital, 369 Fulham Road, London, SW10 9NH UK; 3 North West London Pathology, Fulham Palace Rd, London, W6 8RF UK; 4 National Institute for Health Research Imperial Biomedical Research Centre, Imperial College London, Hammersmith Campus, Du Cane Road, London, W12 0NN UK; 5 Liver, Digestive, and Lifestyle Health Research Section, and Organ Transplant, Centre of Excellence, King Faisal Specialist Hospital and Research Centre, Riyadh, Saudi Arabia; 6 Division of Gastroenterology and Hepatology, Weill Cornell Medicine, New York, NY USA

**Keywords:** Gastritis, Peptic ulcer, Antimicrobial resistance [MeSH], Antimicrobial stewardship [MeSH]

## Abstract

**Background:**

*Helicobacter pylori (H. pylori)* is a Gram-negative bacterium and common cause of gastritis. Antimicrobial treatment typically involves two agents and is prescribed empirically however therapy can be complicated by drug allergies or previous, unsuccessful regimens. Recent data from Europe suggests rising resistance to commonly used agents but contemporary data relevant to UK populations, particularly following the COVID-19 pandemic is limited. This study aimed to report susceptibility testing results in refractory cases of *H. pylori* infections to evaluate local resistance patterns, inform treatment strategies, and compare findings with data from the European Registry on *H. pylori* management.

**Methods:**

A retrospective multi-centre cohort study was conducted between September 2018 and September 2023 at North West London Pathology (London, UK), a central laboratory operating through a hub and spoke model, to assess extended antimicrobial susceptibilities in gastric biopsy samples from patients with refractory *H. pylori* infection. Antimicrobial susceptibilities were assessed using minimum inhibitory concentration methods as per contemporaneous European Committee on Antimicrobial Susceptibility Testing guidelines. Results were compared with European data.

**Results:**

A total of 193 individual isolates were identified. Mean resistance rates were low for tetracycline (2.3%) and amoxicillin (7.3%), moderately low for rifampicin (14.0%), moderate for levofloxacin (27.3%) and high for metronidazole (82.7%) and clarithromycin (75.5%) across the study period. Levofloxacin had a trend of increasing susceptibility (*p =* 0.10) and rifampicin of increasing resistance (*p* = 0.31) throughout the study. Resistance rates were significantly higher for the non-naïve North West London cohort compared with the European non-naïve cohort for metronidazole (*p <* 0.001), amoxicillin (*p <* 0.01), and clarithromycin (*p* = 0.02).

**Conclusion:**

These findings emphasize the necessity of tailored treatment approaches, informed by regional susceptibility patterns. As antimicrobial resistance continues to evolve a proactive and adaptive approach to treatment strategies remains paramount to effectively treat *H. pylori* infection and mitigate associated clinical and financial burdens.

**Clinical trial:**

Not applicable.

**Supplementary Information:**

The online version contains supplementary material available at 10.1186/s12879-025-12236-z.

## Background

*Helicobacter pylori* (*H. pylori*) is a Gram-negative bacterium and is the most common infective cause of atrophic or chronic gastritis and other gastrointestinal diseases, including peptic ulcers, gastric lymphoma and gastric carcinoma [1]. *H. pylori* infects up to 35% of the UK population, causing substantial clinical and financial burden [2].

Current UK clinical practice, as reflected in the National Institute for Health and Care Excellence (NICE) guideline published in 2014, recommends first-line treatment for *H. pylori* infection with a 7-day course of amoxicillin combined with either clarithromycin or metronidazole, alongside a proton pump inhibitor. However, we recognize that more recent international guidelines, including the Maastricht VI/Florence consensus report (2022) recommend longer treatment durations (typically 14 days) and suggest alternative regimens based on updated evidence regarding efficacy and resistance patterns [13]. Despite these evolving recommendations, the 7-day triple therapy remains widely prescribed within the UK healthcare system, and thus forms the basis for much of the antimicrobial resistance data and clinical practice evaluated in this study. These antibiotics should be taken twice daily and should always be prescribed in combination with a proton-pump inhibitor (PPI). For patients who are still symptomatic following this initial treatment, a subsequent 7-day course of a PPI plus amoxicillin and the alternative of clarithromycin or metronidazole (whichever not used first-line) is recommended; further options, such as regimens including a tetracycline or levofloxacin may be considered in those with previous exposure to both clarithromycin and metronidazole [3, 4]. The current UK clinical approach suggests that endoscopy and susceptibility testing should be reserved for patients who remain *H. pylori* positive following two courses of antibiotics, or who have limited treatment options due to local high resistance patterns or hypersensitivity [5]. Contemporary UK data is limited and European data, utilizing the same European Committee on Antimicrobial Susceptibility Testing guidelines could therefore be useful if shown to be similar [6]. Recent reporting of a large European study, but excluding the UK, raises concern for rising resistance rates [7]. Whether this data is relevant to UK populations is currently unclear, as is the potential effect of increased use of broad-spectrum antibiotics during the COVID-19 pandemic.

Despite most laboratories offering only limited option susceptibility testing, North West London Pathology, acting as a centralized diagnostic hub, has introduced a wider susceptibility testing algorithm in recognition of rising antibiotic resistance in addition to supporting the large specialist gastroenterology services available in the region. We therefore report a 5-year (2018–2023) retrospective study of regional susceptibility testing of refractory cases of *H. pylori* infection to evaluate contemporary rates of local resistance and inform testing and treatment strategies and compare findings with the data from the European Registry on H. Pylori Management (Hp-EuReg).

## Methods

### Setting

A multi-centre retrospective cohort analysis was conducted between September 2018 and September 2023 for all *H. pylori* culture positive gastric biopsy samples. Operating through a consolidated hub-and-spoke model, the central laboratory at North West London Pathology provides diagnostic and clinical microbiology services to over two million people, seven acute hospital and approximately 280 primary care healthcare facilities. Samples taken from spoke sites undergo homogenous testing for *H. pylori* in line with UK Standards for Microbiology Investigation (UKSMI) [8] and interpretation of antimicrobial susceptibility testing (AST) by calculation of minimum inhibitory concentrations (MIC) conducted with European Committee on Antimicrobial Susceptibility Testing (EUCAST) breakpoints [6]. The Laboratory Information Management System (Sunquest, Tuscon, AZ, USA) was used to identify and extract all *H. pylori* culture positive samples and their respective antimicrobial susceptibility results.

### Laboratory methods

Samples received at the hub laboratory for AST were managed as per the UKSMI guidelines for identification of *H. pylori.* Samples were cultured on 5% Columbia blood agar, New York City agar and selective *H. pylori* agar plates and incubated at 35–37 °C for 10 days in an atmosphere of 5% oxygen with 5–10% CO_2_. [8] *Helicobacter* species were confirmed by Matrix Assisted Laser desorption ionization-time of flight mass spectrometry (MALDI-TOF) [8]. For the duration of the study, *H. pylori* positive samples were tested by MIC methodology using E-test strips for all of amoxicillin, tetracycline, metronidazole and clarithromycin and on an ad hoc basis for levofloxacin and/or rifampicin at request of the responsible clinician. The testing strategy was expanded to routinely include rifampicin and levofloxacin from August 2020 onwards. EUCAST breakpoints appropriate for the year in which the sample was tested were used to interpret susceptibility results (Supplemental Table 1). Susceptibility refers to the ability of an antibiotic to effectively inhibit or kill *H. pylori*, while resistance indicates the bacterium’s ability to survive despite antibiotic treatment.

### Statistical analysis

Analysis of resistance rates for each antibiotic was initially conducted using descriptive statistics (proportion of isolates that were resistant). A χ2 test for trend was used to assess whether there was a linear trend of resistance to each antibiotic over time (by year) from September 2018 to September 2023. Susceptibility rates for individual agents (North West London cohort) were compared by one-way ANOVA with post-hoc Tukey analysis. Comparison of individual antimicrobial mean susceptibilities between like-for-like (non-naïve) North West London and available European data was then conducted using Student *t*-tests. Significance was set at 0.05.

## Results

A total of 193 *H. pylori* culture positive samples were identified between September 2018 and September 2023. Resistance rates were categorized according to commonly used thresholds in antimicrobial resistance surveillance literature, where resistance prevalence below approximately 15% is generally considered low, 15–30% moderate, and above 30% high [14, 15].

**Fig. 1:**
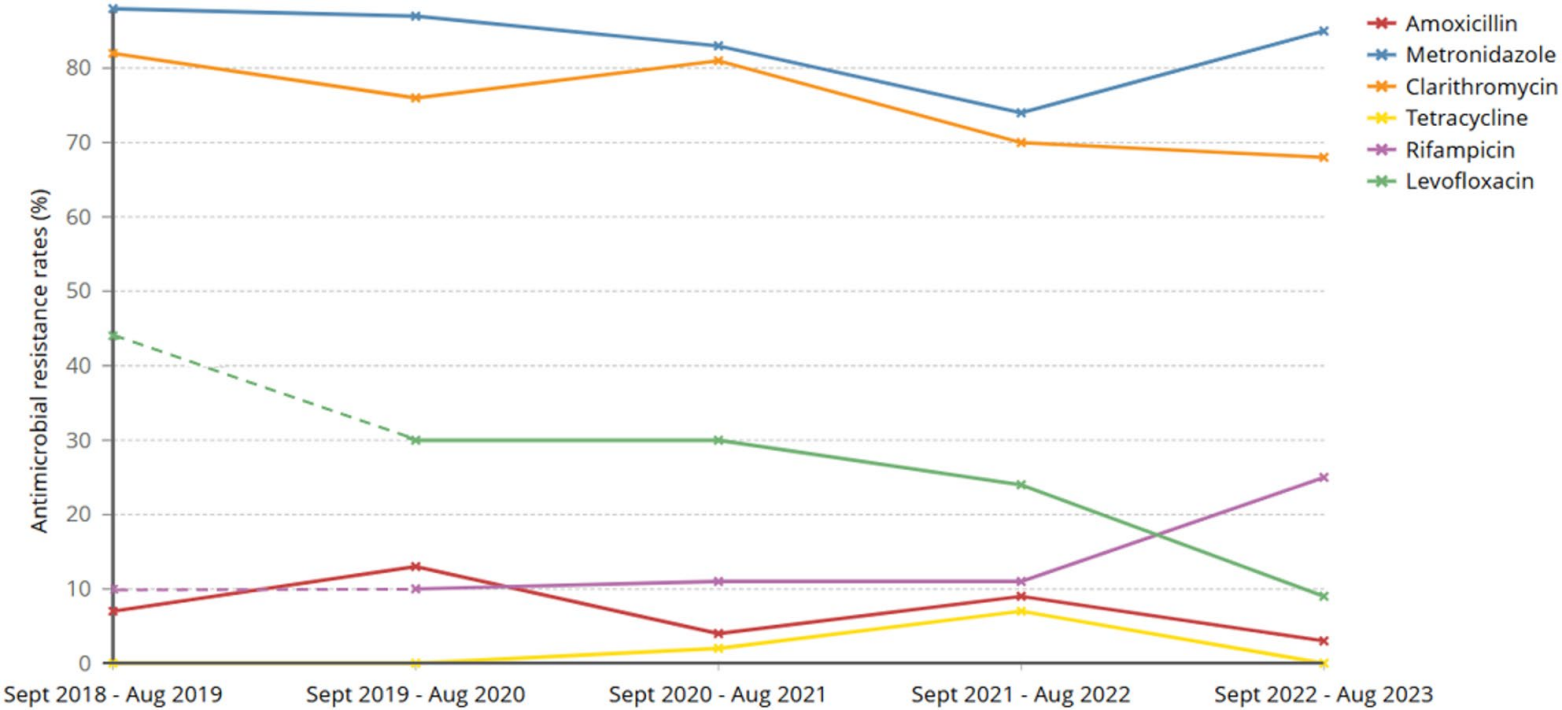
Antimicrobial resistance rates for *H. pylori* between by year between September 2018 and September 2023, North West London, UK. Standard susceptibility testing of *H. pylori* positive cultures was conducted for amoxicillin, clarithromycin, metronidazole and tetracycline throughout the study. Dashed lines represent limited results available from ad hoc testing of rifampicin and levofloxacin on clinician request during that period, switching to continuous lines when testing was routinely expanded to all isolates

Tetracycline (range: 0.0% to 7.0%, *p =* 0.73 from test to trend) and amoxicillin (4.3% to 13.0%, *p* = 0.59) both exhibited low resistance rates throughout the study (Fig. 1). Rifampicin showed moderate-low rates of resistance, with a sharp increase in resistance to rifampicin observed between 2022 and 2023 (10.0% to 25.0%, *p* = 0.31). In contrast, levofloxacin demonstrated a marked decrease in resistance between 2021 and 2023 (44.5% to 8.6%,*p* = 0.10). Clarithromycin showed high resistance rates (82.2% to 67.7%, *p* = 0.50) throughout the study alongside metronidazole (76.0 to 88%, *p* = 0.48) (Fig. 1). When compared with metronidazole or clarithromycin, resistance rates for tetracycline (*p <* 0.001), amoxicillin (*p <* 0.001), rifampicin (*p <* 0.001) and levofloxacin (*p <* 0.001) were all significantly lower.

### Comparison of UK with available non-UK European data

**Fig. 2:**
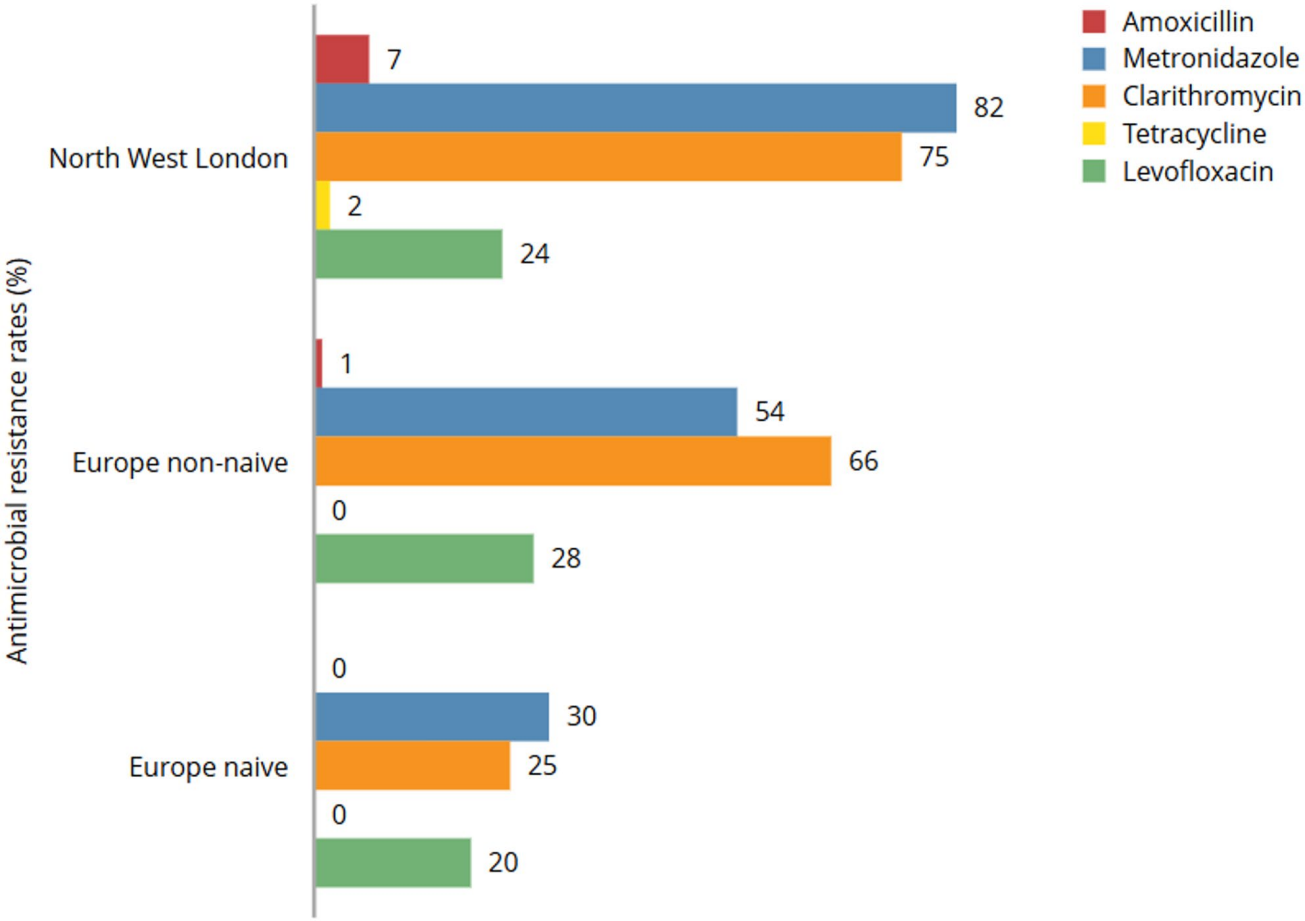
Comparison of *H. pylori* resistance in North West London and the European Registry on *H. pylori* Management (Hp-EuReg). Rifampicin was not included in the European study and so was not included. Data from the European registry represents averaged results across measured susceptibility from 2013–2015 and 2017–2020 while the North West London data is more contemporary, but with some overlap (2018–2023)

Comparison of North West London study data and the European Registry on *H. pylori* Management (Hp-EuReg) [7] shows notable variation. Overall, resistance pattern for North West London were similar to their HpEuReg treatment non-naïve (previous treatment failure) counterpart groups, except for rifampicin which was not reported in the European cohort. Across groups, clarithromycin and metronidazole showed the highest resistance rates with the North West London data reporting the highest rates among the groups (Fig. 2). Resistance rates were significantly different for the non-naïve North West London cohort compared with the European naïve cohort for metronidazole (*p <* 0.001), amoxicillin (*p <* 0.01), and clarithromycin (*p* = 0.02) but not levofloxacin (*p* = 0.31). There were insufficient tetracycline resistant samples to compare.

## Discussion

Our data raises cause for concern for use of antimicrobials as part of both first- and second-line combination therapy options for treating *H. pylori* infection. The study additionally highlights the potential added value of expanded antimicrobial susceptibility testing strategies for this population.

In the UK, paired antimicrobials are delivered in combination with a proton pump inhibitor as part of *H. pylori* treatment strategies. Both first and second-line choices involve either clarithromycin or metronidazole with them being delivered together for patients with a documented penicillin allergy. For context, recent studies in the UK have shown up to 15.7% of patients self-report a penicillin allergy, [9] increasing the likelihood of patients being treated first line with a combination therapy to which we have found high rates of resistance for both drugs. Further UK data in naïve patients could help understand this impact further, yet the HpEuReg data would suggest considerable risk of treatment failure is likely to remain [7]. 

Tetracycline emerged as the most consistently susceptible antimicrobial against *H*. *pylori*, with resistance ranging from 0 to 6.98% across the study. This suggests tetracyclines remain a viable option in those requiring repeated treatments for infection, in keeping with current UK second-line guidance for those requiring re-treatment [10]. In this scenario, doxycycline is often paired with amoxicillin which also demonstrated low overall resistance rates, supporting the use of this combination in refractory cases of *H. pylori* infection in our population. Some variability across the study period in susceptibility rates however, emphasizes the importance of periodic reassessment of treatment strategies, informed by local resistance data, to align with the evolving landscape of bacterial resistance.

While early results suggested comparatively low resistance rates for rifampicin testing, the study noted a trend of rising resistance (10.0% to 25.0%), suggesting the need for caution and further testing in areas where this agent may be utilised. Levofloxacin showed the most significant trend, with decreasing resistance rates (44.5% in 2018 to 8.6% in 2023). During this time antimicrobial stewardship programmes have seen reduced fluoroquinolone prescribing, in line with safety alerts from organisations such as the Medicines Health Regulation Authority, [11] alongside increasing susceptibility rates [12]. This intervention may explain in part the considerable downward trend in resistance rate for levofloxacin.

The European Registry study seeks to provide a scoping situational awareness of *H. pylori* resistance rates across Europe, but does not include any UK specific data. This study constitutes the single largest audit of *H. pylori* sensitivities in the UK to date, with our findings for metronidazole and clarithromycin showing a similar trend but significantly higher levels of resistance in treatment non-naïve patients. This may be in part due to differing prescribing practices or potentially due to inclusion of post-COVID-19 data in the North West London cohort. Regional variations of resistance rates seen across each participating country highlights the importance of understanding local resistance rates in order to best inform treatment options for these populations.

Despite being the largest UK report to date, this study is limited by its regional focus to the region of North West London and the findings might not be generalizable to other UK regions with potentially different antimicrobial resistance patterns. Additionally, pairing of results with community antimicrobial use was not possible, any conclusions around specific treatment strategy failures. Understanding resistance rates among naïve patients would have further added value for informing current antimicrobial guidelines beyond options for those with refractory infections.

We acknowledge that the comparison between our North West London dataset (2018–2023) and the European Registry data (2013–2015, 2017–2020) spans different time periods, which may limit direct comparability. However, some temporal overlap exists, and the comparison still provides valuable regional insights, particularly since UK-specific data are lacking in the European registry. Differences in antimicrobial prescribing practices, as well as potential impacts from the COVID-19 pandemic, may also contribute to variations in resistance patterns between cohorts. By clarifying these temporal differences and contextual factors, we aim to guide readers in interpreting the comparative findings with appropriate caution.

### Summary

The findings of this study emphasize the necessity of tailored treatment approaches, informed by regional susceptibility patterns, to optimize patient outcomes. As antibiotic resistance continues to evolve, a proactive and adaptive approach to treatment strategies (including utilisation of a broadened antimicrobial susceptibility testing strategy), remains paramount to effectively treat *H. pylori* infections, minimise relapses/ multiple-treatment rounds, and mitigate the associated clinical and financial burdens.

## Electronic supplementary material

Below is the link to the electronic supplementary material.

Supplementary Material 1

## Data Availability

Data requests can be made to the corresponding author, as long as data sharing agreements are in place.

## Abbreviations

AMR: Antimicrobial resistance
ANOVA: Analysis of Variance
AST: Antimicrobial susceptibility testing
AZ: Arizona
EUCAST: European Committee on Antimicrobial Susceptibility Testing
Hp-EuReg: European Registry on H. pylori Management
MALDI-ToF: Matrix Assisted Laser desorption ionization-time of flight mass spectrometry
MIC: Minimum inhibitory concentrations
NHS: National Health Service
NICE: National Institute for Health and Care Excellence
NWLP: North West London Pathology
UK: United Kingdom
UK SMI: United Kingdom Standards for Microbiology Investigation
USA: United States of America
