# Supplementary material for: Extended susceptibility testing for refractory Helicobacter pylori infection: regional testing should guide antimicrobial decision making
Source: BMC Infect Dis. 2025 Dec 16;26:101. doi: 10.1186/s12879-025-12236-z (PMC12821947; doi:10.1186/s12879-025-12236-z)
Supplement: Supplementary file 1 — Supplementary Material 1 [file 12879_2025_12236_MOESM1_ESM.docx]

**Supplementary data**

| Antimicrobial | Amoxicillin | | Levofloxacin | | Clarithromycin | | Tetracycline | | Metronidazole | | Rifampicin | | |
| --- | --- | --- | --- | --- | --- | --- | --- | --- | --- | --- | --- | --- | --- |
| EUCAST MIC Guidance | S(<) | R(>=) | S(<) | R(>=) | S(<) | R(>=) | S(<) | R(>=) | S(<) | R(>=) | S(<) | R(>=) |  |
| Vers 8.1 (May 16 - Dec 31 2018) | 0.125 | 0.125 | 1 | 1 | 0.25 | 0.5 | 1 | 1 | 8 | 8 | 1 | 1 |  |
| Vers 9.0 (Jan 1 - Dec 31 2019) | 0.125 | 0.125 | 1 | 1 | 0.25 | 0.5 | 1 | 1 | 8 | 8 | 1 | 1 |  |
| Vers 10.0 (Jan 1 - Dec 31 2020) | 0.125 | 0.125 | 1 | 1 | 0.25 | 0.5 | 1 | 1 | 8 | 8 | 1 | 1 |  |
| Vers 11.0 (Jan 1 - Dec 31 2021) | 0.125 | 0.125 | 1 | 1 | 0.25 | 0.5 | 1 | 1 | 8 | 8 | 1 | 1 |  |
| Vers 12.0 (Jan 1 - Dec 31 2022) | 0.125 | 0.125 | 1 | 1 | 0.25 | 0.5 | 1 | 1 | 8 | 8 | 1 | 1 |  |
| Vers 13.0 (Jan 2 - Jun 29 2023) | 0.125 | 0.125 | 1 | 1 | 0.25 | 0.25 | 1 | 1 | 8 | 8 | 1 | 1 |  |
| Vers 13.1 (June 29 - ongoing) | 0.125 | 0.125 | 1 | 1 | 0.25 | 0.25 | 1 | 1 | 8 | 8 | 1 | 1 |  |

**Supplementary table 1**: Historical EUCAST breakpoint tables for interpretation of MICs and zone diameters for *H. pylori* in use between September 2018 and September 2023.
